# Methods To Identify Aptamers against Cell Surface Biomarkers

**DOI:** 10.3390/ph4091216

**Published:** 2011-09-19

**Authors:** Agnes Cibiel, Daniel Miotto Dupont, Frédéric Ducongé

**Affiliations:** 1 CEA, DSV, I^2^BM, Service Hospitalier Frédéric Joliot (SHFJ), 4 place du général Leclerc, 91401 Orsay, France; E-Mail: agnescibiel@gmail.com (A.C.); 2 INSERM U1023, 4 place du général Leclerc, 91401 Orsay, France; 3 Université Paris Sud, 4 place du général Leclerc, 91401 Orsay, France; 4 Department of Molecular Biology, Aarhus University, Gustav Wieds Vej 10C, 8000 Aarhus C, Denmark; E-Mail: dmd@mb.au.dk (D.M.D.)

**Keywords:** aptamers, SELEX, cell surface biomarkers, methods

## Abstract

Aptamers are nucleic acid-based ligands identified through a process of molecular evolution named SELEX (Systematic Evolution of Ligands by Exponential enrichment). During the last 10-15 years, numerous aptamers have been developed specifically against targets present on or associated with the surface of human cells or infectious pathogens such as viruses, bacteria, fungi or parasites. Several of the aptamers have been described as potent probes, rivalling antibodies, for use in flow cytometry or microscopy. Some have also been used as drugs by inhibiting or activating functions of their targets in a manner similar to neutralizing or agonistic antibodies. Additionally, it is straightforward to conjugate aptamers to other agents without losing their affinity and they have successfully been used *in vitro* and *in vivo* to deliver drugs, siRNA, nanoparticles or contrast agents to target cells. Hence, aptamers identified against cell surface biomarkers represent a promising class of ligands. This review presents the different strategies of SELEX that have been developed to identify aptamers for cell surface-associated proteins as well as some of the methods that are used to study their binding on living cells.

## 1. Introduction

A large number of medical biomarkers are expressed at the surface of human cells or infectious pathogens such as viruses, bacteria, fungi or parasites. The composition and dynamics of the cell surface determine how a cell or a pathogen can interact with its environment and are crucial to send and receive chemical signals, to transport metabolites, ions, or larger molecules, to attach to neighboring cells and the extracellular matrix, *etc.* In the context of disease, a mutation, deletion or over-expression of cell surface proteins is associated with many pathological states and membrane proteins are currently the target for more than half of the approved drugs [1]. Hence, there is a high demand for specific ligands against cell surface targets for fundamental research, but also for diagnosis, monitoring and treatment of diseases. So far, most of these ligands have been developed against proteins whereas only a few exist against carbohydrates and lipids. Two types of ligands have been developed: (1) small molecule drugs which are predominantly designed to bind the intracellular catalytic domain of membrane proteins, and (2) peptide-based ligands or antibodies which are designed to bind both intracellular and extracellular domains.

Among the existing ligands, antibodies are extensively used to profile the expression of cell surface biomarkers and to study their role. Such antibodies have led to the development of the cluster of differentiation (CD) nomenclature initially used for phenotyping cells of the immune system but now extended to many other cell types (http://www.hcdm.org/MoleculeInformation/tabid/54/Default.aspx). However, it can be challenging to identify antibodies recognizing membrane proteins as they exist in their natural environment on the cell surface. Indeed, it is difficult to present membrane proteins to the immune system in their native conformation and for many such targets standard immunization approaches so far have been applied with limited success. Hence, whereas the predicted number of human transmembrane proteins is very high (∼13,000), antibodies have been identified against only 364 CD antigens.

**Figure 1:**
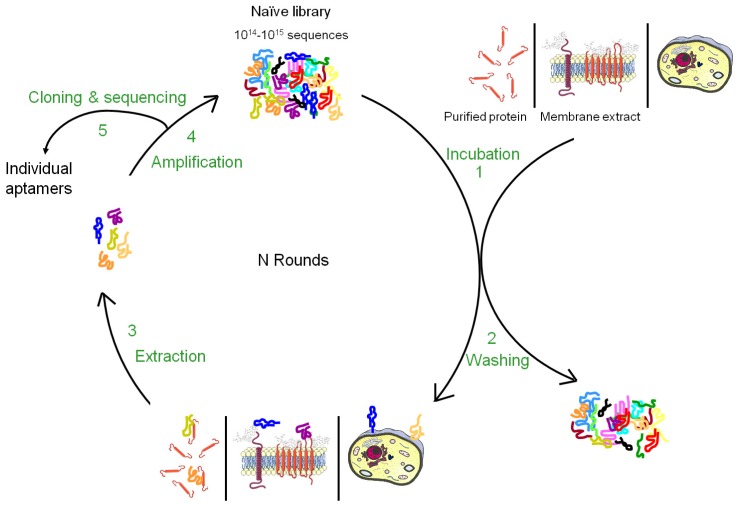
The general principle of the SELEX procedure for a cell surface biomarker. A random pool of oligonucleotide candidates is incubated with a target (purified cell surface biomarker, membrane extract or whole living cell or organism) (1). Sequences which do not bind the target are removed by different partitioning methods (ex: affinity chromatography, filtration, centrifugation) (2). Bound sequences are eluted (ex: urea, EDTA, competition with a ligand) (3) and amplified by PCR (or RT-PCR and *in vitro* transcription in the case of RNA libraries) (4). The selected pool can then enter a new round of selection. During these repetitive rounds of selection, the population evolves towards the sequences with the best affinity for the target. At the end of the process, sequences are cloned and sequenced to identify the aptamers (5).

Nucleic acid-based ligands, named aptamers, appear as appealing new ligands in this field and more than one hundred aptamers have been selected against cell surface targets during the past 10-15 years. Aptamers are generated by a molecular directed evolution approach from a library of 10^14^-10^15^ oligonucleotides containing a region of random base composition [2,3]. This technique is usually named SELEX (Systematic Evolution of Ligands by EXponential enrichment) and consists of repetitive cycles of selection and amplification (Figure 1). During each cycle, oligonucleotides with affinity for a desired target are retained and amplified, leading to their enrichment in the pool which is finally sequenced to identify the aptamers. Since 1990, this strategy has been used to identify aptamers against a wide variety of targets from small molecules to peptides, proteins, nucleic acid-based structures (for reviews, see [4,5]). In many cases, aptamers have been shown to present high specificity and affinity as well as inhibitory or modulatory activity towards their targets [6]. Moreover, they seem to lack immunogenicity and can be chemically modified in order to improve their stability against nucleases, modify their pharmacokinetics or allow labelling. Due to their unique advantages, aptamers have been used in several applications from basic to applied research. For instance, aptamers have been used to study natural interactions between RNA and proteins, to regulate gene expression, to develop biosensors, to purify specific molecules, to inhibit the function of a protein and to develop drugs (for reviews, see [7-10]).

**Figure 2:**
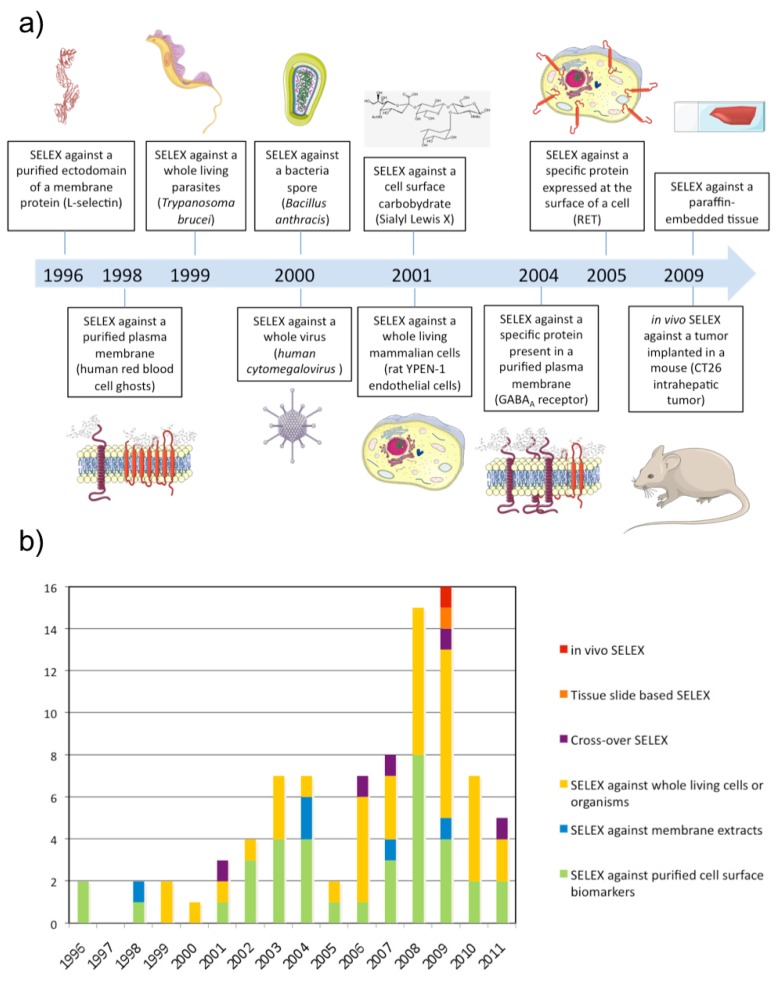
**(a)** Development of new methods of SELEX to identify aptamers against cell surface biomarkers. **(b)** The use of different SELEX strategies over time.

Aptamers recognize the three-dimensional structure of their targets with high specificity and the affinity is often highly dependent on the chosen conditions applied during the selection. It is therefore very important to perform the SELEX against the native conformation of the target and as much as possible in a near natural environment. Such conditions are easily provided for soluble targets but more difficult to achieve for membrane proteins whose structures are inextricably linked to their inclusion in lipidic bi-layers as well as their interaction with the intra- and extracellular matrix. Although several aptamers have been successfully selected against purified cell surface carbohydrates or recombinant ectodomains of membrane proteins, the three-dimensional structure of a purified cell surface biomarker can be rather different from the one found in the context of a plasma membrane. Therefore, aptamers selected against purified domains of membrane proteins are sometimes unable to bind the natural protein expressed at the cell surface. To bypass this drawback, several new methods of SELEX have been developed to allow aptamer selection under conditions as close as possible to the natural situation (Figures 1 and 2). The present review describes the different strategies that have been developed to select aptamers against cell surface biomarkers.

## 2. SELEX Directed against Purified Cell Surface Biomarkers

The first SELEX against a membrane protein was performed against L-Selectin by NeXstar Pharmaceuticals Inc. (Boulder, CO, USA) in 1996 [20]. The L-selectin is a calcium-dependent cell surface lectin that is constitutively expressed on most leukocytes and mediates their adhesion to endothelial cells through interaction with cell-specific carbohydrates. A pool of nuclease-stabilized 2′-aminopyrimidine RNA library was selected at 4 and 22 °C against a human L-selectin-Ig chimera immobilized on Protein A-Sepharose beads. After extensive washing, bound oligonucleotides were eluted with 5 mM EDTA. This SELEX identified aptamers with high affinity at 4 and 22 °C but they had much lower affinity at 37 °C. To isolate aptamers with improved thermal stability, the same group performed the same SELEX at higher selection temperatures (either 22 or 37 °C) using a DNA library [11]. These results highlight that aptamers have to be selected as close as possible to physiological conditions (*i.e.* 37 °C) in order to be potentially usable *in vivo*.

Two years later a SELEX was performed against the full length extracellular domain of CD4 produced from a mammalian expression system and immobilized on beads [12]. To remove sequences with ability to bind to other sites than CD4 in the selection matrix, the library was in each round of the selection pre-exposed to beads lacking CD4, and furthermore, two types of beads were used during selection. The first six rounds were performed against biotinylated CD4 captured on beads coated with streptavidin. Subsequently, another six rounds were performed against CD4 captured onto beads coated with a monoclonal anti-CD4 antibody. Interestingly, the selected aptamers were not only able to bind the recombinant ectodomain of CD4 but also the entire protein expressed on the surface of a mouse T cell line after transfection. In contrast, no measurable binding was observed for the non-transfected cell line lacking the expression of CD4. Hence, aptamers selected against a purified ectodomain of a membrane protein may also be able to recognize the protein in its native environment. Similar SELEX experiments have been performed successfully against other types of membrane proteins including G Protein Coupled-Receptors (GPCRs), Receptor Tyrosine Kinases (RTKs), and Tumour Necrosis Factor (TNF) receptors (see Table 1). In addition, similar strategies have also been applied to select aptamers against proteins from the surface of bacteria and parasites as well as from the envelope or capsid of viruses (see Table 1).

A few aptamer selections have targeted cell surface-associated carbohydrates which represent an important class of cell surface biomarkers as well. The first anti-carbohydrate aptamer was selected against Sialyl Lewis X (sLeX) which is known to be a ligand for the selectin proteins and to be over-expressed at the surface of several types of tumor cells [13]. The selected aptamers had a high affinity (*K_D_* around 10^−9^ to 10^−11^ M) for their target and for similar Lewis group sugars. In contrast, it demonstrated a 100 times lower affinity for unrelated sugars such as lactose. As carbohydrates are often anionic, this could represent a limitation for the selection of aptamers which are themselves negatively charged. To solve this problem, chemically modified aptamers have been developed containing cationic protonated amino groups at the C5 position of thymidines. This strategy was applied successfully to identify aptamers against sialyllactose which is strongly anionic [14]. As for “classical” SELEX against purified soluble proteins, different methods have been used to partition the sequences with affinity for the target from the rest of the pool (see Table 1 and Gopinath *et al.* for a review of these different methods [15]).

In most cases, the selection of aptamers has been performed against the ectodomain of proteins immobilized on a support (magnetic beads, plastic plate or SPR biosensor). The production and purification of a membrane protein ectodomain is often much easier than it is for the whole protein. Furthermore, ectodomains have a higher chance of conserving their proper folding outside the lipid bi-layer. However, in some cases, aptamers identified using this strategy was unable to bind their target in its native form and environment on the cell surface (Table 1 [16-18]). For instance, aptamers selected against the ectodomain of the EGFRvIII protein produced in *E. coli*, were unable to bind the same target produced from an eukaryotic system possessing post-translational modifications [18]. This work demonstrates that the glycosylation state of a protein is a crucial aspect to consider when using recombinant purified protein as a target for SELEX. However, using the glycosylated purified ectodomain is still not a guaranty for obtaining aptamers to the native form. For instance, we found in our group that aptamers selected against the glycosylated ectodomain of the RET^C634Y^ transmembrane receptor were unable to bind their target on the surface of cells although the purified ectodomain was confirmed to bind its natural ligands (GDNF and GFRα1) [16,19]. These results demonstrate that, in some cases, aptamers selected against purified ectodomain proteins may be unable to bind the same target expressed on the surface of cells, even if the purified ectodomain displays a glycosylation pattern and functionality similar to the native protein. To solve this problem, new SELEX procedures have been developed.

## 3. SELEX Directed against Membrane Compartments

In order to select aptamers against membrane proteins in a more physiological environment, SELEX has been performed directly against purified plasma membranes (see Table 2). However, in this case, aptamer selection is conducted towards a mixture of targets present in the plasma membrane. The first SELEX against such a complex mixture of targets was performed using human red blood cell (RBC) ghosts in 1998 [50].

After 25 rounds of selection using a filtration method to recover the sequences bound to RBC membranes, aptamers with high affinity for RBCs were identified. UV-crosslinking experiments demonstrated that this kind of SELEX results in aptamers against several different membrane proteins. However, the drawback of such an approach is that the target of each aptamer at least initially is unknown. To direct the SELEX against a target of interest, specific elution of aptamers using natural ligands of the target has been proposed. This approach was used to select aptamers against several receptors of the nervous system using membrane extracts of brain homogenates from rats [51], *T. Californica* electric organ [52] or a HEK293 cell line expressing the GluR2 glutamate receptor channel [53] (Table 2). During the selections, bound aptamers were eluted using a ligand of the receptor after removal of unbound aptamers by filtration. In addition to selecting aptamers that are specific for a target, this strategy also resulted in aptamers that were potent competitors of the natural ligands of the receptors.

## 4. SELEX Directed against Whole Living Cells, Bacteria, Viruses and Parasites

Although proteins in plasma membrane extracts are in a more physiological environment compared with purified ectodomains, it is well known that the plasma membrane is a dynamic system composed of membrane constituents that are in close contact with each other as well as with the intra- and/or extra-cellular matrix. Therefore, several groups have gone one step further and applied SELEX directly on a whole living and functional biological system (see Table 3). This kind of SELEX was performed for the first time against *Trypanosoma brucei* parasites and *Bacillus anthracis* spores in 1999 [55,56]. In both cases, the partitioning of bound from unbound sequences was done by centrifugation of parasites or bacteria spores. Interestingly, the SELEX against *Trypanosoma brucei* resulted in only a few types of aptamers in spite of the numerous potential targets present on the surface of the parasite suggesting the existence of dominant epitopes. Aptamers were predominantly selected against a target specifically localized in the flagellar pocket of the trypanosome while no aptamer was selected against the VSG protein, which is the most abundant polypeptide on the trypanosome surface. In 2000, SELEX was performed against the human cytomegalovirus using filtration [57]. In that case, aptamers were predominantly selected against the glycoprotein B and H, which represent the most abundant and exposed envelope proteins of the virus.

In 2001, SELEX was performed for the first time on a whole living mammalian cell line [58]. During the SELEX, the library was counter-selected in each round against N9 microglial cells before selecting against rat YPEN-1 endothelial cells. As the two cell types were non-adhering cells, unbound sequences were removed by centrifugation. The counter-selection was performed to favour aptamers against targets whose expression is linked to the endothelial phenotype. The pool was fluorescently labeled during the SELEX, which allowed the monitoring of enrichment of cell-binding aptamers using flow cytometry. After sequencing, most of the individual sequences tested showed affinity for the YPEN-1 EC cell line by flow cytometry, and some of them could bind microvessels in cryostat tissue sections of rat brain glioblastomas. Since then, several aptamer selections have been conducted with living cells and the strategy has been named cell-SELEX. While centrifugation is the partitioning method of choice for non-adherent cells, in the case of adherent cells, gentle plate washing is used. However, recently another method was described by Raddatz *et al.* who used fluorescence-activated cell sorting (FACS) to perform the partitioning of bound from unbound sequences [59]. The study demonstrated that FACS was a powerful method for selecting aptamers against living Burkitt lymphoma B cells particularly because it allows the elimination of dead cells, which usually display high non-specific binding for nucleic acids.

As mentioned previously for SELEX against membrane compartments, SELEX against a living biological system often also results in the identification of aptamers for an unknown target. It can be difficult to identify the unknown target of an aptamer, but it opens a new avenue for the application of SELEX within biomarker identification and cell phenotyping. For example, the subtractive cell-based method described for the SELEX against the YPEN-1 endothelial cell line allowed the identification of aptamers against targets that are differentially expressed between different cell types. Furthermore, aptamers have also been selected with ability to distinguish cells on the basis of differentiation state [60], distinguish cancer cells or virus infected cells from normal cells of the same origin [61-63] and discriminate for instance different lineages of hematopoietic cancers [64,65] (see Table 3). For such aptamers, target identification can lead to the identification or validation of cell specific biomarkers. The strategy has been named AptaBiD for Aptamer-facilated Biomarker Discovery [66]. Several protocols have been used to isolate and identify the target protein of aptamers selected using cell-SELEX [58,67-70]. Basically, affinity chromatography is performed using biotinylated aptamer pre-incubated with a membrane protein extract, a total protein extract, or alternatively with living cells followed by cell lysis. Bound protein is subsequently recovered and separated or not by SDS-PAGE electrophoresis before being identified by mass spectrometry.

## 5. SELEX Directed against a Specific Biomarker in a Complex Mixture

Different strategies have been developed to select aptamers against a specific cell surface target using living organisms or cells. As previously described for SELEX against purified plasma membranes, displacement of aptamers using ligands of known biomarkers has been used, for example, to isolate aptamers against cell surface proteins of *T. cruzi* which interact with host cellular matrix components [93]. Cell surface proteins of the extracellular matrix bind to the parasite surface and affect host cell invasion. For the selection of aptamers against the parasite, aptamer elution was performed using a mix of proteins from the extracellular matrix including laminin, fibronectin, heparan sulfate and thrombospondin in order to isolate high affinity aptamers with ability to compete with host cell matrix molecules for binding to *T. cruzi* cell surfaces. Furthermore, to select aptamers specific for the infective form of the parasite, a counter-selection step using non-infective epimastigote surfaces was applied in round seven of the SELEX experiment.

In our group, we developed a strategy for selection of aptamers to specific cell surface targets using transfected cells which over-express a protein of interest. To validate the approach, we selected aptamers against transfected PC12 cells over-expressing the human mutated receptor tyrosine kinase RET (RET^C634Y^), which is constitutively activated in its dimeric form. In each round, we performed counter-selection steps against mock-transfected PC12 cells and PC12 cells over-expressing a monomeric activated mutant of RET (RET^M918T^) [16]. Using this strategy we were able to identify an aptamer that inhibits RET dimerization-dependent signaling pathways induced either by its natural ligand (GDNF) or by the C634Y activating mutation. However, the aptamer did not distinguish the dimeric form of RET from the monomer form in spite of counter-selection using RET^M918T^ expressing cells. Furthermore, it represents a minority of the selected sequences (2 out of 67 sequences). Similar strategies have also been used to identify aptamers against transforming growth factor-β type III (TbRIII) and the HCV-E2 envelope glycoprotein [73,80]. Alternatively, aptamers against HER-2 were selected using SK-BR-3 known to over-express the membrane protein, while counter-selecting using the same cell line treated with HER-2 siRNA [81].

Another strategy involves an immuno-precipitation step and was developed to select aptamers against the Toll-like receptor 2 [82]. In this study, Toll-like receptor 2 fused to an Fc fragment was transiently expressed in HEK293T cells. The DNA library was incubated with whole cells before lysis or directly to cell lysates and the sequences specifically bound to the receptor recovered using protein A beads.

## 6. Cross-Over SELEX

In comparison to SELEX against purified targets, the selection of aptamers from a complex mixture of targets, as for example in cell-SELEX, is more difficult to direct towards specific proteins, however, in the latter case the membrane proteins are in an environment more close to the native situation. To compile the advantages of both types of SELEX methods, cross-over strategies have been developed, in which, shifting between steps of SELEX against a purified protein and cells expressing the protein are performed. An aptamer for Tenascin-C was obtained using this kind of strategy [95]. After nine rounds of cell-SELEX, two rounds were performed against the purified protein improving the affinity of the pool for the protein 50-fold. In our group, we have showed that the same strategy allowed higher specific enrichment of aptamers for RET than cell-SELEX alone, but that the selected aptamers using pure whole-living cell-SELEX display a better apparent *K_D_* [19].

More recently, cross-over SELEX has been used to obtain aptamers directed against CD16α protein [96]. Nine rounds were performed against purified protein and then five rounds against cells overexpressing the protein of interest. More than 25 aptamers were found to bind the purified protein whereas only two of them bound CD16α on recombinant Jurkat or NK cells. One of these aptamers was already present at the end of the selection against the purified protein whereas the other was present only in the population selected against both purified protein and cells overexpressing it.

In an aptamer selection for the P2X2 receptor, another strategy was applied, involving shifting between selection against plasma membrane extracts containing the membrane protein and selection against the purified form inserted in immobilized artificial membranes (IAMs) [97]. IAMs have been used previously to immobilize various types of transmembrane proteins including transporters, carriers and receptors. In all cases, the proteins retained the ability to bind their ligands and a setup with IAMs could be used to determine their pharmacological properties. After three initial cycles of SELEX using plasma membrane preparations containing P2X2 receptor, the pool was loaded on a P2X2-IAM column for chromatographic selection of RNA molecules with binding affinity to the P2X2 receptor. One round of IAM chromatography SELEX was able to improve the affinity of the pool to the receptor by 300%.

## 7. Tissue Slide-Based SELEX

Recently, SELEX has been performed directly against cancerous tissue [98]. Li *et al.* performed SELEX against paraffin-embedded tissue sections from infiltrating ductal carcinomas and counter-selection against the adjacent normal tissue from the same case. An advantage of this method is that it's possible to select aptamers to all fractions of tissue including extracellular matrix, membrane components and intracellular targets. One aptamer, BC15, was identified to specifically recognize breast cancer cells from clinical tissue sections of different pathological types and breast cancer cell lines. The target of this aptamer was identified to be the intracellular protein hnRNP A1.

## 8. *In Vivo* SELEX

As mentioned previously, cell-SELEX can be performed to identify aptamers against specific cell surface biomarkers of a disease without any prior knowledge of the markers. However, the question remains whether cells in culture are relevant models for a disease. With the purpose of adapting the SELEX procedure to a complete physiological environment, SELEX has recently been performed *in vivo* to select aptamers recognizing intrahepatic colorectal cancer metastasis [99]. A 2′F-Py RNA library was injected intravenously in a mouse bearing a previously implanted hepatic tumour. Liver tumours were collected and RNA was subsequently extracted and amplified. The new population of 2′F-Py RNA was re-injected in a new mouse and the process repeated several times. Interestingly, evolved RNA pools demonstrated higher affinity for a tumour protein extract than a normal colon cell protein extract and the population of round 14 was cloned and sequenced. Interestingly, the target of one aptamer was determined to be p68, an intracellular RNA helicase known to be upregulated in colorectal cancer.

## 9. Methods To Study the Binding of Aptamers on Living Organisms or Cells

Whatever the SELEX method used, after sequencing the pool, the binding of individual aptamer clones has to be evaluated on living organisms or cells. During this evaluation, the affinity of the aptamer can be determined but also its specificity for its target, since it is found in a mix with many other biomarkers at the cell surface. Several strategies have been presented for measuring the binding of aptamers to living cells. Basically, aptamers are incubated with cells at different concentrations and bound aptamer quantified after extensive washing. Using Scatchard analysis, the *K_D_* and Cmax (apparent concentration of target at the cell surface) can be measured for a specific cell line. When cells are adherent, a possibility is to incubate cells with 5′-[32P]-radiolabeled aptamer and following a washing step, detect bound aptamer by scintillation counting [16,95]. When cells are non-adhering, many studies apply fluorescent aptamers to perform similar analyses by flow cytometry. The latter technique has also been applied as a tool for monitoring the progress of cell-SELEX experiments [62,64,65]. The technique can also be used with adherent cells, but cells have to be put in suspension before flow cytometry analysis. However, the presence of some proteins on plasma membranes may be affected by the procedures associated with detachment of adhering cells from the solid support of the culture plate. For instance, Li *et al.* demonstrated that the aptamer for Tenascin-C was not a good probe for flow cytometry analysis using U251 glioma cells whereas it labeled the same cells efficiently after adhesion using microscopy [100]. Tenascin-C is an extracellular matrix glycoprotein and the authors hypothesized that the protein may be removed and degraded by the process of detachment using trypsin which was performed prior to flow cytometry analysis.

## 10. Conclusions and Perspectives

During the past 10-15 years, there have been a growing number of reports describing the identification of aptamers against cell surface-associated targets. During this time, several different strategies for performing SELEX have also been developed to enable selection of aptamers of particularly membrane proteins under near-physiological conditions. These advanced strategies increase the probability of selecting aptamers against membrane targets in their native form and natural environment on the cell surface. However, these new SELEX methods are not always straightforward compared with classical SELEX methods against purified targets. Hence, some pitfalls of cell-SELEX have been described that can cause failure of the selection process. For instance, the presence of dead cells during selection can capture non-specifically a high number of oligonucleotides [59,104], counter-selection is not always efficient [16,19], and purification of DNA single strands using streptavidin beads can be imperfect [105]. Furthermore, *in vivo* SELEX in small animals and tissue slide-based SELEX have not yet been reproduced two years after having been published for the first time, eventhough these SELEX methods are particularly promising. Nevertheless, all these new methods are expected to be improved over the next decade and will lead to the selection of many aptamers.

Cell surface targeting aptamers can potentially be used for several applications. For instance, aptamers that bind to membrane proteins involved in disease can often inhibit or activate their targets leading to the development of new drugs. For example, a neutralizing anti-nucleolin aptamer is currently in phase IIb clinical trials for the treatment of Acute Myeloid Leukemia (AML) in combination with cytarabine [101]. Additionally, aptamers targeting cell surface proteins have been successfully used as targeting moieties to deliver contrast agents, nanoparticles encapsulating drugs, or siRNA to specific tissues *in vivo* [102,103]. Furthermore, they are being used within the development of diagnostic assays in fields such as cancer, infectious disease, food safety and bioterrorism. Finally, a more recent area of interest for aptamer selections against membrane proteins is the use of methods such as cell-SELEX within cell surface biomarker discovery and cell phenotyping. As a consequence, selections of aptamers against cell surface biomarkers have a high potential use not only for fundamental research, but also for diagnosis, monitoring and treatment of diseases.

## Figures and Tables

**Table 1:** SELEX directed against purified cell surface biomarkers.

**Chemistry**	**Target**	**Number of cycles**	**Partition**	**Binds native protein**	**Reference**
***Mammalian cell surface markers***					
2′NH2-py RNA	L-selectin	14	Affinity chromatography	Yes	[20]
DNA	L-selectin	17	Affinity chromatography	Yes	[11]
2′F-Py RNA	CD4	15	Affinity chromatography	Yes	[12]
RNA	Sialyl Lewis X	17	Affinity chromatography	Yes	[13]
2′F-Py RNA	PSMA	6	Affinity chromatography	Yes	[21]
2′F-Py RNA	NTR	7	Affinity chromatography	Yes	[22]
RNA	HER3	15	Membrane filtration/gel shift assay	Yes	[23]
2′F-Py RNA	CTLA-4	9	Membrane filtration	Yes	[24]
RNA	RANK	7	Affinity chromatography	ND	[25]
DNA	RGD/fibonectine	5 + 5	Affinity chromatography/titration	Yes	[26]
Modified DNA	Sialyllactose	13	Affinity chromatography	ND	[14]
RNA	TLR3	7	Membrane filtration	ND	[27]
DNA	MUC1	10	Affinity chromatography	ND	[28]
2′F-Py RNA	AMPA receptor	8	Membrane Filtration	Yes	[29]
DNA	DC-SIGN	11	Titration plate	Yes	[30]
2′F-Py RNA	VCAM-1	12	Affinity chromatography	No	[17]
2′F-Py RNA	OX40	11	Affinity chromatography	Yes	[31]
2′F-Py RNA	4-1BB	12	Affinity chromatography	Yes	[32]
RNA	TfR	9	Affinity chromatography	Yes	[33]
DNA	TfR	6	Affinity chromatography	Yes	[33]
2′F-Py RNA	EGFR	12	Affinity chromatography	No	[18]
DNA	MUC1/MUC1GalNac/GalNac	10	Affinity chromatography/titration	Yes	[34]
Thio-DNA	CD44-HABD	10	Affinity chromatography	Yes	[35]
2′F-Py RNA	EpCAM	12	Affinity chromatography	Yes	[36]
***Virus surface markers***					
2′F-Py RNA	gp120	9	Membrane filtration	ND	[37]
2′F-Py RNA	Gp120	5	Surface plasmon resonance	Yes	[38]
DNA	HA	3	Affinity chromatography	Yes	[39]
DNA	HA1	11	Affinity chromatography	Yes	[40]
2′F-Py RNA	SARS-CoV N	9	Affinity chromatography	ND	[41]
RNA	HBsAg	12	Membrane filtration	Yes	[42]
***Bacterial surface markers***					
RNA	PilS	8	Affinity chromatography	Yes	[43]
RNA	BipD/BopE/BPSL2748		Membrane filtration	ND	[44]
DNA	LPS	5	Affinity chromatography	Yes	[45]
DNA	a-PDGA	5	Affinity chromatography	ND	[46]
DNA	PA	8	Membrane filtration	ND	[47]
***Parasite surface markers***					
DNA	KMP-11	10	Affinity chromatography	ND	[48]
2′F-Py RNA	DBL1α	8	Affinity chromatography	Yes	[49]

**Table 2:** SELEX directed against membrane compartments.

**Chemistry**	**Membrane extract**	**Targeted protein**	**Number of cycles**	**Partition**	**Reference**
***Mammalian membranes***					
DNA	RBC ghosts	ND	25	Filtration	[50]
2′F-Py RNA	rat brain membranes	GABA_A_ receptor	12	Filtration	[51]
2′F-Py RNA	*T califonica* electric organ	nAChR	9	Filtration	[52]
RNA	HEK293 expressing GluR2 subunit	AMPA receptor	14	ND	[53]
***Bacterial membranes***					
DNA	*Salmonella enterica* serovars	ND	7	Filtration	[54]

**Table 3:** SELEX directed against whole living systems.

**Chemistry**	**Selection**	**Counter-selection**	**Targeted or identified targets**	**Number of cycles**	**Reference**
***Mammalian Cells***					
DNA	YPEN-1	N9	Pigpen	8	[58]
DNA	B lymphoma cells	No	ND	10	[71]
DNA	Differenciated PC12	PC12	ND	6	[60]
DNA	U251	No	Tenascin-C	21	[67]
2′F-Py RNA	CHO-kinin B1 receptor	No	Kinin B1 receptor	8	[72]
2′F-Py RNA	PC12-MEN2A	PC12/PC12-MEN2A	RET	15	[16]
2′F-Py RNA	CHO-TbRIII	CHO	TbRIII	11	[73]
DNA	aMSCs	No	ND	11	[74]
DNA	CCRF-CEM	Ramos	PTK7	20	[65,70]
DNA	Ramos	No	mmunoglobin heavy mu chain	23	[69,75]
DNA	SAOS-2 osteoblasts	No	ND	10	[68]
DNA	MEAR	BNL	Non-muscle myosin	16	[62]
DNA	NCI-H69	NCI-H661	ND	25	[76]
DNA	EPCs	No	ND	10	[77]
DNA	iDC (mDC)	mDC (iDC)	Several (see reference)	10	[66]
DNA	Burkitt lymphoma cells	No	ND	10	[59]
DNA	A549	HLAMP	ND	25	[78]
DNA	HL60	NB4	ND	16	[64]
2′F-Py RNA	U87MG	T98G	ND	14	[79]
DNA	CT26-E2	CT26	Virus Glycoprotein E2	13	[80]
DNA	Infected A549	Non-infected A549	ND	13	[63]
RNA	SKBR3	SKBR3/siHER-2/MDAMB231	HER-2	20	[81]
DNA	HEK293T-TLR2-Fc	No	TLR2	7	[82]
DNA	Vaccinia virus-infected HeLa	Non-infected HeLa	Hemagglutinin	20	[61]
2′F-Py RNA	HET-SR-1	HET-SR	ND	10	[83]
DNA	Tov-21G/CAOV-3	HeLa	ND	22	[84]
DNA	DLD-1/HCT 116	HCT 116/HT-29	ND	16	[85]
DNA	NIH3T3	IL-17RA-deficient NIH3T3	IL-17RA	12	[86]
***Viruses***					
2′NH2-Py RNA	Human cytomegalovirus	No	Glycoprotein B and H	16	[57]
RNA	Influenza A panama virus	Influenza A Aichi Virus	Haemagglutinin	10	[87]
***Bacteria***					
DNA	*B. anthracis* spores	No	ND	4	[55]
DNA	*M. Tuberculosis* H37Rv	*M. Tuberculosis* BCG	ND	10	[88]
DNA	*L. Acidophilus*	No	S-layer protein	8	[89]
DNA	*S. aureus*	*Streptococcus/S. epidermidis*	ND	5	[90]
DNA	*C. Jejuni*	Non relevant mix	ND	10	[91]
DNA	*P. aeruginosa*	*S. Maltophilia/A. Baumannii*	ND	16	[92]
***Parasites***					
RNA	*T. brucei*	No	ND	12	[56]
2′F-Py RNA	*T. cruzi* trypomastigote	Epimastigote	Several (see reference)	8	[93]
2′NH2-Py RNA	*T. brucei*	No	ND	13	[94]
